# Determinants and prevalence of symptomatic dengue fever among adults in the Central Region of Burkina Faso: a hospital-based cross-sectional study

**DOI:** 10.1186/s12879-023-08932-3

**Published:** 2024-01-02

**Authors:** Jean Claude Romaric Pingdwindé Ouédraogo, Sylvain Ilboudo, Tegwindé Rebeca Compaoré, Prosper Bado, Mathieu Nitiéma, Wendlasida Thomas Ouédraogo, Salfo Ouédraogo, Mohamadi Zeba, Alix Tougma, Geoffroy Gueswindé Ouédraogo, Noufou Ouédraogo, Sylvin Ouédraogo, Léon Gueswendé Blaise Savadogo

**Affiliations:** 1 Laboratoire de Développement de Médicament (LADME), Ecole Doctorale Sciences de La Santé, Université Joseph Ki-Zerbo, Ouagadougou, Burkina Faso; 2 Laboratoire de Recherche-Développement de Phytomédicaments Et Médicaments (LR-D/PM), Institut de Recherche en Sciences de La Santé (IRSS), Ouagadougou, Burkina Faso; 3 International Research Laboratory - Environnement, Santé Et Sociétés (IRL 3189, ESS), CNRST/CNRS/UCAD/UGB/USTTB, Ouagadougou, Burkina Faso; 4 Laboratoire de Recherche en Maladies Infectieuses Et Parasitaires (LR/MIP), Institut de Recherche en Sciences de La Santé (IRSS/CNRST), Ouagadougou, Burkina Faso; 5 Centre de Recherche Biomoléculaire Pietro Annigoni (CERBA), Ouagadougou, Burkina Faso; 6 Direction Régionale de la Santé du Centre, Ouagadougou, Burkina Faso; 7 Laboratoire de Recherche Sur Le Patrimoine Et Le Développement Durable (LR/PDD), Institut Des Sciences Des Sociétés (INSS/CNRST), Ouagadougou, Burkina Faso; 8 Université Joseph KI-ZERBO/Centre Universitaire de Ziniaré, Ziniaré, Burkina Faso; 9 Institut Supérieur des Sciences de la Santé, Université NAZI BONI, Bobo-Dioulasso, Burkina Faso

**Keywords:** Dengue fever, Malaria, Coinfection, Seroepidemiological study, Symptoms, Markers, Burkina Faso

## Abstract

**Background:**

Dengue fever (DF) is a significant public health concern in Burkina Faso, particularly in the Central Region, previously endemic for malaria. However, limited research has focused on dengue prevalence and associated factors among adult febrile patients in this region. This study aimed to estimate the prevalence of symptomatic dengue fever among adults and identify the sociodemographic and clinical determinants of the disease.

**Methods:**

A seroepidemiological cross-sectional study was conducted in the Central Region of Burkina Faso, through a three-stage sampling. Five health facilities, one from each of the region five districts, were purposively selected. Febrile patients aged 16 and older, suspected of having dengue, were included in the study, after consenting. Bivariate analyses and multivariate binary logistic regression were done at a 5% confidence level.

**Results:**

A total of 637 patients between the ages of 16 and 90 years were included. Most of the participants were females (58.71%). Most dengue cases resided in Arrondissement 4 (59.62%), or were present in the Arrondissement 4 at daytime during the previous days (51.92%). 52.90% of the participants knew of dengue. Dengue prevalence was estimated at 8.16% (95% CI: 6.16%-10.57%). The most frequent markers for dengue were immunoglobulins M detected in 4.40% (2.94%-6.29%), followed by Antigen NS1 at 4.24% (95% CI: 2.81%-6.11%). The Antigen NS1 marker was associated with myalgia (*p* = 0.024), vomiting (*p* < 0.001), hemorrhagic manifestations (*p* = 0.001), and anorexia (*p* < 0.001). Staying at Arrondissement 4 (vs staying at Saaba) during daytime (aOR = 2.36 95% CI: 1.03–5.45; *p* = 0.044) significantly increased the odds of dengue. Dengue cases were about 3 times more likely to have vomited (aOR = 2.99 95% CI: 1.58–5.64; *p* = 0.001). Participants knowing of dengue (aOR = 0.53 95% CI: 0.29–0.98; *p* = 0.042) and those coinfected with malaria (aOR = 0.28 95% CI: 0.14–0.57; *p* < 0.001) instead had reduced odds of dengue.

**Conclusion:**

The study revealed a relatively high prevalence of symptomatic dengue fever among adults in the Central Region of Burkina Faso in 2022. These findings emphasize the need for continuous surveillance and targeted control measures. The low coinfection of dengue and malaria warrants further investigation.

## Introduction

Dengue fever (DF) is a viral disease that poses a significant global health threat, particularly in subtropical and tropical climates, usually semi-urban and urban settings. It is estimated that dengue fever threatens about half of the population worldwide. According to the World Health Organization (WHO), the burden of dengue has increased from 505 430 cases in 2000 to 5.2 million cases in 2019 [1]. The disease is now endemic in 100 countries, with the Americas, South-East Asia, and Western Pacific regions being the most affected areas [1]. However, estimates give Africa a similar burden to the Americas [2–4]. A meta-analysis of previous studies indicated that dengue prevalence among febrile patients in Africa stands at 8.4% (95% confidence interval (CI): 3.7–14.4), 10.8% (95% CI: 3.8–20.6), and 24.8% (95% CI: 13.8–37.8) for the Ribonucleic acid (RNA), immunoglobulins M and immunoglobulins G, respectively [5].

According to the “Level of Risk” classification established by the CDC, United States of America, DF is frequent/continuous in Burkina Faso [2]. Number of studies have confirmed its endemicity with epidemics notified in 2013, 2016, and 2017 [2, 6–8]. Patients detected with Antigen NS1 and Immunoglobulin M proportioned 11% and 4%, respectively during the period from 2014 to 2017 in the Central Region of Burkina Faso [9, 10]. More recently, dengue was featured as the most frequently neglected tropical disease in hospitals and Medical Centres and the fifth most frequent in primary healthcare facilities in 2020 [11]. Evidence shows that all the four serotypes (DENV-1, DENV-2, DENV-3 and DENV-4) are circulating in Burkina Faso [9, 10, 12]. However, these estimates could not be accurate, as dengue is underreported and misdiagnosed in Africa as other illnesses like malaria or zika [3, 12, 13]. Healthcare facilities could be overwhelmed in the event of a massive influx of dengue cases, calling for accurate estimations of symptomatic DF for programmatic purposes.

Despite the documented prevalence of DF in the region, limited research has focused on estimating the burden of symptomatic dengue fever among febrile adult in Burkina Faso, while adults showed higher frequencies of mortality related to DF [14]. From a single laboratory-based data, dengue and malaria was negatively associated with the coinfection at 1.4% [7]. However, systematized studies focused on non-malarial participants that did not allow assessing the prevalence of dengue fever in this setting previously endemic for malaria [9, 10]. Thus, this study estimated the prevalence and identified the determinants of dengue fever among symptomatic adult patients in the Central Region in Burkina Faso.

## Methods

### Aim

This study aimed to estimate the prevalence of symptomatic dengue fever among adults in the Central Region of Burkina Faso and determine the sociodemographic and clinical factors associated with.

### Study design

This study is part of an overall study aiming to estimate the prevalence of symptomatic and asymptomatic DF among adults in Burkina Faso over 2022–2023, through hospital and household-based data collections. Within this research, we conducted a hospital-based cross-sectional study. Data collection took place between September and November 2022 at various health facilities.

### Study setting

The study was conducted in the Central Region of Burkina Faso, covering and including the capital city, Ouagadougou, and six rural municipalities: Koubri, Saaba, Pabré, Komsilga, Komki-Ipala, and Tanghin-Dassouri [15]. The region has a tropical north Sudanese climate, with a dry season from November to May and a rainy season from June to October. In 2019, the population of the Central Region was 3,032,668, making it the most populous region in the country and including 62.4% adults [15, 16]. In 2020, the Central Region reported approximately 70% of dengue cases in the country [11].

**Fig. 1:**
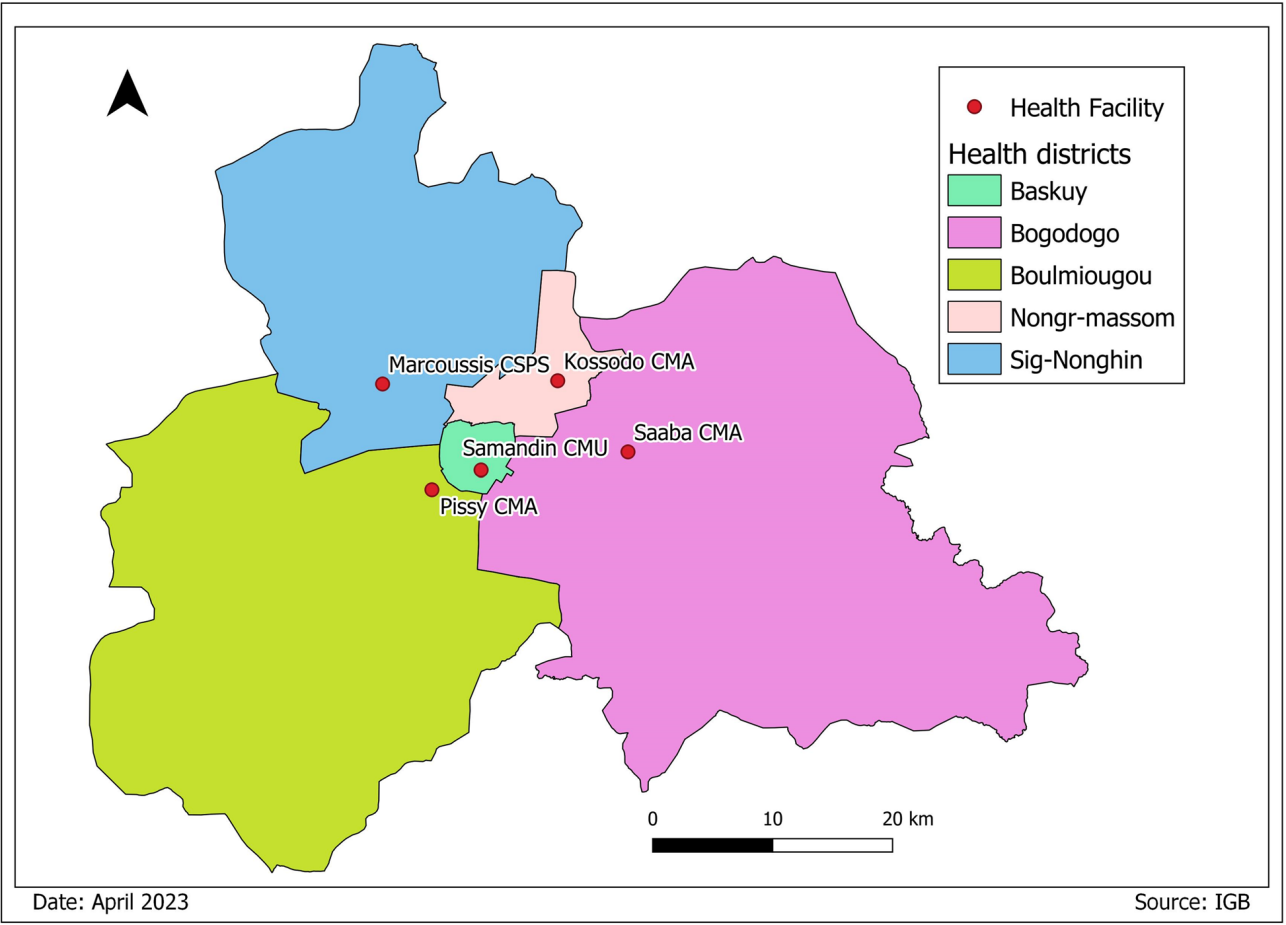
Map showing the 5 health districts of the Central region—Burkina Faso

The region is divided into five health districts: Baskuy, Nongr Massom, Sig-Nonghin, Bogodogo, and Boulmiougou (Fig. 1). Each health district includes several primary healthcare centres (CSPS), Medical Centres (CM), Medical Centres with Surgical Antenna (CMA), Urban Medical Centres (CMU) and/or private health facilities. In effect, the health system is pyramidal, with the district at the bottom organized around the CMA and including the CSPS, CM and/or CMU, then the regional hospital centres, and at the top the university hospital centres and national hospitals.

### Participants

The study targeted individuals 16 years of age or older, suspected of having dengue fever based on the following criteria: having a fever (≥ 38.5 °C) and presenting at least two of the following symptoms or signs in the last five days: headache, retro-orbital pain, myalgias, arthralgias, skin rash, bleeding manifestations, or shock syndrome. A suspected case that turned positive to the dengue rapid diagnostic test was a dengue probable case [17].

Mentally debilitated individuals were excluded from the study.

### Sample size estimation

The formula for a single proportion estimation was used to determine the required sample size [18]:

$${\text{n}}\ge \frac{{Z}_{1-\propto /2}^{2} \times p(1-p)}{{e}^{2}}$$

-p: anticipated prevalence of symptomatic dengue fever cases;

-${Z}_{1-\alpha /2}$: percentage standard deviation corresponding to the two-sided significance level. For $a=5\%, {Z}_{1-\alpha /2}=1.96$.

-e: precision of 4%.

The prevalence of dengue among symptomatic populations in Africa was estimated at 10.8% (95% CI 3.8–20.6) [5]. This prevalence was higher than 10%, then an anticipated prevalence of 50% was used to get a maximum sample size with a precision of 4%. The sample size was 601, further adjusted by a 10% non-response rate with this formula (n = $\frac{{n}_{0}}{1-{n}_{r}}$) to get 668 febrile participants.

### Sampling technique

We performed a three-stage sampling. At the district level, all five health districts in the Central Region were included in the study. Then, for each district, the facility that reported the highest number of dengue cases in 2021 in the district was purposively selected. The selected health facilities were Samandin CMU (Baskuy district), Saaba CMA (Bogodogo district), Pissy CMA (Boulmiougou district), Kossodo CMA (Nongr-massom district), Marcoussis CSPS (Signonghin district). At the individual level, participants who agreed to participate were consecutively included until the required sample size was reached.

### Questionnaire and data collection

Data collection involved administering a structured questionnaire and conducting tests. The questionnaire collected information on independent variables related to participants’ sociodemographic characteristics, knowledge of dengue fever and clinical features. Dependent variables were the status of dengue fever (Positive/negative) and the marker presence of dengue (NS1 Antigen, Immunoglobulin M and G), based on the RDTs. The presence of NS1 Antigen was considered as an acute dengue, the Immunoglobulins M and G respectively as a recent or ancient dengue. But participants with only a positive IgG could be experiencing a secondary infection. Participants with AgNS1 and IgM were considered as acute primary infections. Those exhibiting simultaneously AgNS1 and IgG were suspected of having acute secondary dengue infections.

After the questionnaire administration, participants were tested for dengue and malaria using rapid diagnostic tests (RDT). The WONDFO Dengue NS1/IgG/IgM test (Guangzhou Wondfo Biotech Co., Ltd, China), an antigenic and serological test, was used for dengue. According to the manufacturer, the sensitivity and sensibility for WONDFO Dengue NS1 Antigen Test were 92.22% and 100%, respectively. With the WONDFO Dengue IgG/IgM Antibody Test, the sensitivity was 97.30% and the specificity 98.18%. The antigenic kit SD Bioline Malaria Ag P.f (SD Standard Diagnostics, INC., Republic of Korea) tested for malaria. These RDTs were done following the procedures given by the manufacturers.

Data were collected electronically in smartphones using Kobo Toolbox, through a face-to-face interview. They were further exported into an Excel file for analyses.

### Statistical analyses

Univariate, bivariate, and multivariate analyses were done with STATA/IC 16.1 (StataCorp LLC, College Station, Texas 77845 USA).

Dengue fever prevalence among these symptomatic cases was estimated with 95% confidence intervals. The dependent variable was then symptomatic dengue fever, referring to the participant being negative or positive to dengue RDT. Independent variables were presented with mean ± standard deviation (SD) or median (interquartile range (IQR)) for the quantitative variables, or absolute and relative frequencies for the qualitative variables. They were presented for the overall sample, dengue cases, and non-positive participants.

Bivariate analysis, using a Chi2 of Pearson’s test or a Fischer’s Exact test, assessed the association between the symptoms/signs and acute dengue, recent or ancient dengue.

Further, a binary logistic regression was done to identify the determinants of symptomatic dengue among suspected cases (*p*-values of odds ratios [OR]) and the level of influence (OR). Factors significant at 10% and those pertinent in the univariate logistic regression were included in the multivariable analysis.

## Results

### Sociodemographic features of the participants

A total of 637 participants were included in the study. Details of their characteristics are shown in Table 1. Most dengue cases were reported in the Kossodo CMA (61.54%).

**Table 1:** Distribution of participants characteristics

**Characteristics**	**Overall *n* = 6**37 **n (%)**	**Probable dengue cases** ***n* = 5**2 **n (%)**	**Non-dengue patients** ***n* = 585** **n (%)**
**Health facilities**			
Kossodo CMA	242 (37.99)	32 (61.54)	210 (35.90)
Saaba CM	225 (35.32)	9 (17.31)	216 (36.92)
Samandin CMU	151 (23.70)	9 (17.31)	142 (24.27)
Pissy CMA	13 (2.04)	2 (3.85)	11 (1.88)
Marcoussis CSPS	6 (0.94)	0 (0.00)	6 (1.03)
**Age (years)**	30.57 ± 12.54	30.29 ± 12.54	30.60 ± 12.55
	27 (21–36)	26 (20.5–36.5)	27 (21–36)
	16–90	16–75	16–90
**Sex**			
Female	374 (58.71)	29 (55.77)	345 (58.97)
Male	263 (41.29)	23 (44.23)	240 (41.03)
**Education level**			
No education	135 (21.19)	10 (19.23)	125 (21.37)
Primary level	94 (14.76)	10 (19.23)	84 (14.36)
Secondary level	281 (44.11)	20 (38.46)	261 (44.62)
Tertiary level	127 (19.94)	12 (23.08)	115 (19.66)
**Marital status**			
Single	338 (53.06)	29 (55.77)	309 (52.82)
Married	281 (44.11)	22 (42.31)	259 (44.27)
Widowed/Divorced	18 (2.83)	1 (1.92)	17 (2.91)
**Main occupation**			
Student	176 (27.63)	14 (26.92)	162 (27.69)
Trader	124 (19.47)	8 (15.38)	116 (19.83)
Private employee	116 (18.21)	12 (23.08)	104 (17.78)
Housewife	77 (12.09)	7 (13.46)	70 (11.97)
Public servant	51 (8.01)	3 (5.77)	48 (8.21)
Unemployed	35 (5.49)	2 (3.85)	33 (5.64)
Oher occupations	58 (9.11)	6 (11.54)	52 (8.89)
**Residence**			
Saaba	221 (34.69)	10 (19.23)	211 (36.07)
Arrondissement 4	219 (33.75)	31 (59.62)	184 (31.45)
Arrondissement 1	104 (16.33)	6 (11.54)	98 (16.75)
Arrondissement 6	25 (3.92)	2 (3.85)	23 (3.93)
Arrondissement 10	21 (3.30)	0 (0.00)	21 (3.59)
Other residences	51 (8.01)	3 (5.77)	48 (8.21)
**Main place last 7 days**			
Home	320 (50.24)	24 (46.15)	296 (50.60)
Workplace	214 (33.59)	21 (40.38)	193 (32.99)
School	63 (9.89)	5 (9.62)	58 (9.91)
Other places	40 (6.28)	2 (3.85)	38 (6.50)
**Place during daytime**			
Arrondissement 4	211 (33.12)	27 (51.92)	184 (31.45)
Saaba	209 (32.81)	10 (19.23)	199 (34.02)
Arrondissement 1	115 (18.05)	7 (13.46)	108 (18.46)
Arrondissement 6	21 (3.30)	2 (3.85)	19 (3.25)
Arrondissement 10	19 (2.98)	1 (1.92)	18 (3.08)
Other places	62 (9.73)	5 (9.62)	57 (9.74)
**Number of family contacts**	5 (3–7)	5 (3–7)	5(3–7)
	1–26	1–20	1–26
**Number of adult contacts**	3 (2–4)	3 (1–4)	3 (2–4)
	1–25	1–10	1–25
**Knowledge of dengue**			
Yes	337 (52.90)	21 (40.38)	316 (54.02)
No	300 (47.10)	31 (59.62)	269 (45.98)
**Knowledge of the germ**			
Don’t know	592 (92.94)	48 (92.31)	544 (92.99)
Virus	45 (7.06)	4 (7.69)	41 (7.01)
**Knowledge of transmission ways**			
Don’t know	257 (40.35)	18 (34.62)	239 (40.85)
Mosquito bite	380 (59.65)	34 (65.38)	346 (59.15)

The median age was 27 (21–36), ranging from 16 to 90 years. Females were predominant among both dengue cases (55.77%) and non-dengue participants (58.97%). The distribution of education level, marital status, primary occupation, and place stayed during the last 7 days was similar among participants, probable dengue, and non-dengue cases.

Most positive cases resided in Arrondissement 4 (59.62%) and Saaba (19.23%). Similarly, most dengue cases spent their daytime at Arrondissement 4 (51.92%) and Saaba (19.23%). The median number of family and adult contacts was similar for the dengue patients and non-dengue participants.

### Dengue fever seroprevalence

Out of the 637 participants, 52 individuals tested positive for dengue RDT, resulting in an estimated prevalence of 8.16% (95% CI: 6.16%-10.57%). Among the positive cases, 4.24% (95% CI: 2.81%-6.11%) showed signs of acute dengue infection indicated by the presence of Antigen NS1. The most common type of dengue observed was recent dengue infection, with 28 patients [4.40% (95% CI: 2.94%-6.29%)] testing positive for Immunoglobulins IgM. Nine participants [1.40% (95% CI: 0.65%-2.67%)] displayed an ancient dengue marker, indicated by the presence of Immunoglobulins IgG. Three (3) individuals exhibited simultaneously AgNS1 and IgM and were considered as acute primary infections. In addition, 2 cases could have a secondary dengue infection with both AgNS1 and IgG.

Malaria prevalence was estimated at 43.17% (95% CI: 39.29%-47.12%), with all 275 positive cases to *Plasmodium falciparum*. The coinfection of dengue fever and malaria was estimated at 2.20% (95% CI: 1.21%-3.66%).

### Signs and symptoms associated with dengue marker type

Certain signs and symptoms were found to be associated with specific markers of dengue fever (Table 2). Myalgia (*p* = 0.024) was significantly associated with acute dengue fever, as well as vomiting (*p* < 0.001), hemorrhagic manifestations (*p* = 0.001), and anorexia (*p* < 0.001). No symptom or sign was associated with recent dengue fever, nor ancient or secondary dengue fever.

**Table 2:** Bivariate analysis showing the symptoms and signs associated with dengue fever markers

Presence of symptoms and signs	Acute dengue fever AgNS1 + (*n* = 24)		Recent dengue fever IgM + (*n* = 28)		Ancient or secondary dengue fever IgG + (*n* = 9)	
	**n (%)**	***p*-value**	**n (%)**		**n (%)**	***p*-value**
**Temperature (> 38.5 °C)**	6 (22.22)	0.728	4 (14.29)	0.467	1 (11.11)	0.517
**Headaches**	26 (96.30)	0.864	28 (100)	0.330	9 (100)	0.586
**Retro-orbital pain**	5 (18.52)	0.966	2 (7.14)	0.121	2 (22.22)	0.753
**Myalgia**	9 (33.33)	**0.024**	11 (39.29)	0.099	2 (22.22)	0.087 [FET]*
**Arthralgia**	17 (62.96)	0.098	16 (57.14)	0.291	4 (44.44)	1 [FET]*
**Nausea**	6 (22.22)	0.943	6 (21.43)	0.975	1 (11.11)	0.439
**Vomiting**	18 (66.67)	** < 0.001**	11 (39.29)	0.299	3 (33.33)	0.850
**Abdominal pain**	8 (29.63)	0.975	6 (21.43)	0.346	3 (33.33)	0.792
**Hemorrhagic manifestations**	3 (11.11)	**0.001** [FET]*	0 (0.00)	1.000 [FET]*	0 (0.00)	1 [FET]*
**Chills**	0 (0.00)	0.273	1 (3.57)	0.889	1 (11.11)	0.283
**Anorexia**	5 (18.52)	** < 0.001**	1 (3.57)	0.799	1 (11.11)	0.342
**Asthenia**	2 (7.41)	0.594	0 (0.00)	0.206	1 (11.11)	0.419
**Dizziness**	3 (11.11)	0.544	2 (7.14)	0.863	1 (11.11)	0.730

^*^FET: Fischer’s Exact test

### Factors associated with dengue fever

The results of the binary logistic regression are presented in Table 3. Based on the univariate analysis, several factors were found to be associated with dengue fever at a significance level of 5%. These factors include the residence, the location where participants stayed during the daytime, specific symptoms (myalgia, vomiting, and hemorrhagic manifestations), as well as malaria**.**

**Table 3:** Sociodemographic and clinical factors associated with dengue fever occurred in the Central Region, Burkina Faso

**Characteristics**	**Univariate binary logistic regression**		**Multivaria**bl**e binary logistic regression**	
	**Crude Odds ratio**	***p*-value**	**Adjusted Odds ratio**	***p*-value**
**Age**	1.00 (0.98- 1.02)	0.864	1.00 (0.97–1.02)	0.756
**Sex**				
Female	1		1	
Male	1.14 (0.64- 2.02)	0.653	1.19 (0.65–2.19)	0.577
**Education level**				
No education	1		-	-
Primary level	1.49 (0.59–3.73)	0.397	-	-
Secondary level	0.96 (0.44–2.11)	0.915	-	-
Tertiary level	1.30 (0.54–3.13)	0.552	-	-
**Marital status**				
Single	1		-	-
Married	0.91 (0.51–1.61)	0.735	-	-
Widowed/Divorced	0.63 (0.08–4.88)	0.656	-	-
**Main occupation**				
Student	1		-	-
Trader	0.80 (0.32–1.96)	0.624	-	-
Private employee	1.34 (0.59–3.00)	0.484	-	-
Housewife	1.16 (0.45–2.99)	0.763	-	-
Public servant	0.72 (0.20–2.62)	0.622	-	-
Unemployed	0.70 (0.15–3.23)	0.649	-	-
Other occupations	1.33 (0.49–3.65)	0.573	-	-
**Residence**				
Saaba	1		-	-
Arrondissement 4	3.55 (1.70–7.45)	**0.001**	-	-
Arrondissement 1	1.29 (0.46–3.66)	0.629	-	-
Arrondissement 6	1.83 (0.38–8.89)	0.451	-	-
Other residences	0.92 (0.25–3.43)	0.898	-	-
**Principal place last 7 days**				
Home	1		-	-
Workplace	1.34 (0.73–2.48)	0.347	-	-
School	1.06 (0.39–2.90)	0.905	-	-
Other places	0.65 (0.15–2.86)	0.568	-	-
**Place during daytime**				
Saaba	1		1	
Arrondissement 4	2.92 (1.38–6.20)	**0.005**	2.36 (1.03–5.45)	**0.044**
Arrondissement 1	1.29 (0.48–3.48)	0.616	1.34 (0.46–3.98)	0.591
Arrondissement 6	2.09 (0.43–10.27)	0.362	2.39 (0.44–13.00)	0.314
Other places	1.59 (0.56–4.53)	0.384	1.44 (0.48–4.33)	0.520
**Number of family contacts**	0.99 (0.92–1.06)	0.782	-	-
**Number of adult contacts**	1.00 (0.91–1.11)	0.952	1.03 (0.92–1.15)	0.581
**Knowledge of dengue**				
No	1		1	
Yes	0.58 (0.32–1.03)	0.062	0.53 (0.29–0.98)	**0.042**
**Knowledge of the germ**				
Don’t know	1		-	-
Virus	1.11 (0.38–3.22)	0.854	-	-
**Knowledge of transmission ways**				
Don’t know	1		-	-
Mosquito bite	1.30 (0.72–2.36)	0.381	-	-
**Difference between dengue fever and malaria**				
No	1		-	-
Don’t know	1.21 (0.46–3.18)	0.696	-	-
Yes	1.39 (0.56–3.47)	0.474	-	-
**Temperature**				
Below 38.5 °C	1		-	-
Above 38.5 °C	0.62 (0.27–1.40)	0.247	-	-
**Presence of signs and symptoms (ref. absence)**				
Headaches	1.71 (0.22–13.05)	0.604	-	-
Retro-orbital pain	0.68 (0.30–1.55)	0.357	-	-
Myalgia	0.49 (0.28–0.88)	**0.017**	0.74 (0.39–1.43)	0.378
Arthralgia	1.71 (0.96–3.05)	0.068	1.53 (0.81–2.89)	0.190
Nausea	1.09 (0.56–2.15)	0.796	-	-
Vomiting	2.48 (1.40–4.40)	**0.002**	2.99 (1.58–5.64)	**0.001**
Abdominal pain	0.88 (0.46–1.66)	0.688	-	-
Hemorrhagic manifestations	17.85 (2.91–109.36)	**0.003**	-	-
Chills	0.44 (0.06–3.31)	0.425	-	-
Anorexia	2.49 (0.91–6.82)	0.077	1.70 (0.57–5.07)	0.337
Asthenia	0.71 (0.17–3.07)	0.652	-	-
Dizziness	0.95 (0.33–2.76)	0.931	-	-
**RDT malaria**				
Negative RDT	1		1	
Positive RDT	0.46 (0.24–0.86)	**0.016**	0.28 (0.14–0.57)	** < 0.001**

LR chi2(13) = 38.52, Pseudo R2 = 0.1069, AIC = 349.69

*n* = 637

*p* = 0.0002

Residing or staying during the daytime at Arrondissement 4 were approximately associated with 4- or 3-times higher odds of having dengue fever, respectively, compared to residing at or visiting Saaba during the day. Similarly, suspected cases who vomited were twice as likely to test positive for dengue, compared to those who did not vomit (*p* = 0.002). Participants with hemorrhagic manifestations had about 18-folds increase chance of having dengue than those with no hemorrhagic manifestations (*p* = 0.003). Conversely, participants with myalgia are less likely to test positive to dengue to the extent of 50%, compared to those without myalgia. Holding all other variables constant, having a malaria RDT positive reduced the odds of dengue infection by about 54%, compared to having a negative result (*p* = 0.016).

Considering the probability of collinearity, only the location where participants stayed during daytime was included in the multivariable analysis, although residence was also significant at 5%. Interestingly, knowledge of dengue became significantly associated with dengue, causing confusion, as it reduces the odds of dengue by 47% (*p* = 0.042). Being positive for malaria still reduced the odds of dengue to the extent of 72% (*p* < 0.001). Staying at Arrondissement 4 during the daytime (compared to Saaba) and vomiting (compared to not vomiting) were associated with an increased chance of dengue by 2.36 times (*p* = 0.044) and 2.99 times (*p* = 0.001), respectively.

## Discussion

This cross-sectional study aimed to estimate the prevalence of symptomatic dengue fever and assess associated factors among adults. It included testing febrile participants seeking care in healthcare facilities in the Central region—Burkina Faso- for dengue and malaria.

### Symptomatic dengue prevalence

The prevalence of dengue fever was relatively high in this study, however similar to previous findings (8.7%) in 2013–2014 among both children and adults in primary healthcare facilities in Ouagadougou [10]. According to Ridde et al*.*, participants age was statistically associated with probable/confirmed dengue, and about 90% of the cases were adults [10]. Similarly, adult dengue cases represented over 80% of the participants in primary healthcare facilities in Ouagadougou between December 2014 and February 2017 [9]. This study does not allow a conclusive long-term evaluation of DF among adults, due to a lack of previous estimations of DF among adults. Further, as it focused on this population, this study should be taken as a basis for future surveillance of adult dengue fever in the Central Region of Burkina Faso.

In this study, acute dengue accounted for 4.24% of the adult participants in 2022, slightly lower than the result found in another study by Bello, who reported 5.4% of acute DF cases among febrile patients of all ages in 2020–2022 with laboratory-based data in Ouagadougou [19]. Regarding recent dengue cases (IgM positive), we recorded 4.40% among the participating population. In an earlier study, acute DF cases accounted for a significantly higher prevalence (11%) among all febrile patients in Ouagadougou between 2014 and 2017 [9]. However, the same study reported a similar percentage of recent dengue cases (4%) than what was found in this study. Variations in marker positivity rates are likely due to differences in study participants and design. Higher proportions of IgM may reflect the delay in seeking care while presenting fever and other symptoms.

### Signs/symptoms associated with dengue markers

Symptoms like fever, body pain, headache, nausea, and rash are frequent with dengue [1]. A previous study found rash and retroorbital pain associated with DF during the outbreak period (September to November 2016) and rash and nausea/vomiting in the non-outbreak period among febrile participants, including children and adults [9]. This study reported only on adults, so differences in symptoms were expected.

In this study, the symptomatologic profile associated with acute dengue infection was specific compared to recent and ancient cases. Acute infections were associated with myalgia, vomiting, anorexia, and hemorrhagic manifestations, while no symptom or sign was related to recent or ancient dengue. These findings can improve clinical diagnosis, particularly when rapid diagnostic tests (RDT) for DF are not readily available.

### Factors associated with dengue fever

Multivariable analysis revealed that being in Arrondissement 4 during the daytime and experiencing vomiting increased the odds of dengue fever. With the other 4 districts of the Central region, Nongr-massom district constituted the main cluster of dengue between 2016–2019 in Burkina Faso [20]. This suggests that Arrondissement 4 could be a hotspot for dengue transmission, as *Aedes* mosquitos bite during the day. In effect, Nongr-Massom district includes 2 of the 3 dams of Ouagadougou and the "Bangr-Weogo" urban park. The "Zone du Bois", a residential area, is also located in Arrondissement 4 with many trees, flowerpots, gardens and swimming pools in the houses and services. Nonetheless, entomological studies are necessary for a more conclusive hypothesis to help implement vector control measures.

This study found that participants with higher knowledge of dengue were less likely to experience dengue. In 2013, when the public discovered dengue fever with the ongoing epidemic, it was called “Palu-dengue” [malaria-dengue in English], as the symptoms or signs of the two diseases are close. They believed that dengue was a new form of malaria. With about 53% of the participants claiming to know about dengue, awareness about the disease has improved in 2022. These improvements could be partially due to community-based interventions for *Aedes* vector control that could have reduced the confusion between malaria and dengue and increased populations awareness of dengue symptoms [21, 22]. These interventions included breeding sites destruction as well as behavior change interventions with messages on mosquito breeding sites identification, and dengue prevention and management. However, efforts are still needed as most dengue cases claimed they did not know of dengue (59.62%) or the causal agent (92.31%). While it was expected that knowledge would likely reduce the risk of dengue fever, it is surprising that coinfection with malaria also significantly reduces the dengue infection.

Participants co-infected with malaria significantly had reduced odds of dengue in both univariate and multivariate analyses. Nevertheless, few studies have been done about malaria-dengue coinfection. A study that pooled 13 studies in 2019 estimated an odds ratio of 0.32 (95% CI: 0.27–0.36) for coinfected cases compared to only malaria cases [23]. These studies were published between 2009 and 2016; three were done in Africa (Nigeria, Tanzania, and Senegal). In addition, *Plasmodium* parasitemia was significantly lower in coinfected cases than in patients with malaria [23]. From a single laboratory data, the coinfection of dengue-malaria was as high as 1.4%, with these diseases negatively associated [7]. Similarly, coinfection was low (2.84%) among febrile patients of all ages in Nigeria, despite the high prevalence of Immunoglobulin M antibodies at 46% [24]. These results were consistent with the estimates in this study (2.20%), specifically among adults. Further studies should focus on estimating the burden of dengue-malaria coinfection in Burkina Faso.

### Limitations

Prevalences found in this study are subject to the specificity and sensitivity of the RDT tests used and the sociodemographic and clinical characteristics [25]. As a hospital-based study, the findings may not represent the true epidemiology of dengue fever in Burkina Faso, as many cases are mild or asymptomatic and do not seek care at healthcare facilities, as well as certain symptomatic cases. Future epidemiological investigations should capture symptomatic dengue patients who do not seek care and include asymptomatic individuals.

## Conclusion

This study provides valuable insights into the prevalence of symptomatic dengue fever among adults in Burkina Faso, particularly in the Central Region. To our knowledge, this study is the first to focus on adults in Burkina Faso, setting a basis for further surveillance of dengue fever. With a high prevalence among adults, symptomatic dengue fever is a public health concern in Burkina Faso, specifically for those residing or spending the daytime in Arrondissement 4. Moreover, malaria reduced the risk of dengue fever, links that need to be further explored.

## Data Availability

The datasets used and/or analysed during the current study are available from the corresponding author on reasonable request.

## Abbreviations

AgNS1: Antigen Nonstructural protein 1
CM: Medical Centre
CMA: Medical Centre with Surgical Antenna
CSPS: Primary healthcare centres
DF: Dengue fever
DENV: Dengue virus
IgG: Immunoglobulins G
IgM: Immunoglobulins M
CMU: Urban Medical Centres
RDT: Rapid Diagnostic Test
